# Use of chest X-ray in the assessment of community acquired pneumonia in primary care – an intervention study

**DOI:** 10.1080/02813432.2020.1794404

**Published:** 2020-07-24

**Authors:** Anna B Moberg, Moa Kling, Jakob Paues, Sven Göran Fransson, Magnus Falk

**Affiliations:** a Kärna Primary Healthcare Centre, Linköping, Sweden; b Kungsgatan Primary Healthcare Centre, Linköping, Sweden; c Department of Infectious Diseases and Department of Biomedical and Clinical Sciences, Linköping University, Linköping, Sweden; d Department of Health, Medicine and Caring Sciences and Department of Radiological Sciences, Linköping University, Linköping, Sweden; e Department of Health, Medicine and Caring Sciences, General Practice, Linköping University, Linköping, Sweden

**Keywords:** Family practice, pneumonia, chest X-ray, antibiotics, general practice, intervention, primary care

## Abstract

**Objectives:**

The aim of this study was to explore if consequent use of chest X-ray (CXR), when the physician is not sure of the diagnosis of pneumonia after clinical examination and CRP-testing, favors a more restrictive prescribing of antibiotics.

**Design:**

This was an intervention study conducted between September 2015 and December 2017.

**Setting:**

Two intervention primary health care centers (PHCCs) and three control PHCCs in the southeast of Sweden.

**Intervention:**

All patients were referred for CXR when the physician´s suspicion of pneumonia was ‘unsure’, or ‘quite sure’ after CRP-testing. Control units managed patients according to their usual routine after clinical examination and CRP-testing.

**Subjects:**

A total of 104 patients were included in the intervention group and 81 patients in the control group. The inclusion criteria of the study were clinically suspected pneumonia in patients ≥18 years, with respiratory symptoms for more than 24 h.

**Main outcome measure:** Antibiotic prescribing rate.

**Results:**

In the intervention group, 85% were referred for CXR and 69% were prescribed antibiotics, as compared to 26% and 77% in the control group. The difference in antibiotic prescribing rate was not statistically significant, unadjusted OR 0.68 [0.35–1.3] and adjusted OR 1.1 [CI 0.43–3.0]. A total of 24% of patients with negative CXR were prescribed antibiotics.

**Conclusion:**

This study could not prove that use of CXR when the physician was not sure of the diagnosis of pneumonia results in lowered antibiotic prescribing rate in primary care. In cases of negative findings on CXR the physicians do not seem to rely on the outcome when it comes to antibiotic prescribing.

**Key Points**
- Routine use of chest X-ray when the clinical diagnosis of pneumonia is uncertain has not been proven to result in lowered antibiotic prescribing rate.
- Physicians do not fully rely on chest X-ray outcome and to some extent prescribe antibiotics even if negative, when community-acquired pneumonia is suspected.
- Chest X-ray is already used in one out of four cases in routine primary care of pneumonia patients in Sweden.

## Introduction

Excessive use of antibiotics has caused an increasing rate of drug-resistance. For instance, globally the prevalence of community-acquired pneumonia (CAP) caused by drug-resistant *Streptococcus pneumoniae*, is 1–7% [1]. The most frequent reason for systemic antibiotic prescribing is respiratory tract infections, which are mainly managed in primary care. Lower respiratory tract infection (LRTI) is one of the most lethal communicable diseases, causing 2.4 million yearly deaths worldwide [2]. LRTIs include acute bronchitis and pneumonia. Usually, acute bronchitis should not be treated with antibiotics, as it is often of viral etiology and thus normally a self-limiting condition [3,4]. Pneumonia, which contributes to 54% of LRTI deaths in all ages worldwide even among young patients, is often of bacterial origin and should therefore be treated with antibiotics [5,6]. To appropriately identify patients with pneumonia is as essential as it is challenging. Over-diagnosing pneumonia results in unnecessary use of antibiotics and, in the long run, bacterial resistance [7]. To date, the recommendations on how to manage pneumonia in primary care differ between countries [8–11]. There are several guidelines and decision algorithms, of which none has proven to reliably distinguish between patients who will benefit from antibiotics and those who will not [12–16]. In a previous study, we showed that the physician´s degree of suspicion correlates well to findings on chest X-ray (CXR) [17]. Although being the gold standard for diagnosing pneumonia, European guidelines do not recommend CXR to be used routinely in the assessment of pneumonia [9–11].

According to the current Swedish guidelines [11], pneumonia should be suspected in case of a generally sick patient with concomitant symptoms such as fever, cough, recent onset fatigue and/or lateral chest pain especially in combination with typical clinical findings such as focally depressed or altered breathing sounds or dullness to percussion, tachypnea and tachycardia. The initial judgement does not include C-reactive protein (CRP)-testing nor CXR examination according to Swedish guidelines. When the diagnosis of LRTI is unclear after clinical examination, CRP-testing might be considered. If still in doubt, the recommendation is either watchful waiting, consideration to refer for CXR or delayed antibiotic prescribing.

We aimed to explore the benefit of CXR examination when the primary care physician was not sure of the pneumonia diagnosis after clinical examination and CRP-testing, and if consequently using it this way can alter the rate of antibiotic prescribing. Our hypothesis was that using CXR in this way would result in a lowered antibiotic prescribing rate.

## Materials and methods

### Participants

Prior to study start, a sample size calculation was made, assuming a statistical power of at least 80% and a significance level of 5%. Data from our previous study gave an assumption of a 40/60 proportion between patients prescribed antibiotics and not. Based on this, approximately 100 patients in each group were required [17].

The study was conducted during regular working hours at five Swedish primary health care centers (PHCCs) between September 2015 and December 2017, when the intended one hundred patients in the intervention group had been recruited. Both general practitioners (GPs) and resident physicians recruited patients. Two of the PHCCs served as intervention units and three as control units. Initially, only two PHCCs participated as control units, but due to a somewhat slow inclusion rate, one more health care center was included from December 2016 and allowed inclusion of patients to continue for the same time interval as the other PHCCs. There was no randomization. The PHCCs serving as intervention units had recently participated in a similar study where CXR was used for all patients, with any degree of suspicion of pneumonia. Therefore, we considered these centers not to be representative as control units. The control units were chosen as they were similar in characteristics, such as demographics, and distance to hospital (<10km). Patients were included consecutively when the doctor suspected pneumonia.

The inclusion criteria were; patient ≥18 years, respiratory symptoms for more than 24 h and any degree of suspicion of pneumonia by the physician, after clinical examination and CRP-testing. Pregnancy, known as chronic obstructive pulmonary disease (COPD) and living at a nursing home, were exclusion criteria. All participants gave written informed consent and the regional ethics review board in Linköping, Sweden approved the study (Dnr 2015/223-31).

### Intervention

The intervention consisted of an advised clinical routine to consistently refer all patients with suspected pneumonia for CXR, unless the physician was ‘sure’ of the diagnosis of pneumonia after clinical examination and CRP-testing. A decision of prescribing antibiotics was made after CXR result or in case of being ‘sure’ of the diagnosis without further investigation. The physicians at the intervention units were instructed by means of an information meeting, in conjunction with written instructions.

At the control units, the physicians were advised to manage the patients with suspected pneumonia after clinical examination and CRP testing, following their usual clinical routine/national guidelines.

### Measurements

When the physicians in the initial consultation with a patient with LRTI suspected pneumonia, they were instructed to document anamnestic data and findings from the clinical examination in a case report form (CRF). Different characteristics and clinical findings such as age, gender, duration of symptoms, smoking habits, body temperature (measured with a digital ear thermometer), and abnormal chest sounds at auscultation, use of antipyretics, and position of the physician were documented. Capillary blood samples for CRP (Quick Read Go™, Orion Diagnostics Oy, Sweden or Alere Afinion™ AS100) were drawn from all participants and results were documented in the CRF. The different devices, used to analyze CRP, had different upper limits ranging from 160 to 200 mg/L. Therefore, values above 160 and below 5 mg/L were set to 160 and 5 mg/L, respectively.

Based on clinical examination and laboratory evaluation, the degree of suspicion of pneumonia was rated by the physicians into one of three degrees; ’unsure’, ‘quite sure’ or ‘sure’ of the diagnosis of pneumonia. The scale was used in a recent study where a strong correlation between the degree of suspicion and radiographic findings was shown [17]. This scale has not been validated in any other way.

When patients were referred for CXR it was executed according to ordinary clinical routine, i.e. frontal and lateral views, within 48 h. Positive radiographic findings were defined as the presence of a new consolidation in the definitive statement. Uncertain answers were considered positive. The radiologist received clinical and anamnestic information and the inquiry was ’infiltrate?’ or ’pneumonia?’ Every examination was initially viewed by the radiologist on duty for a preliminary statement, followed by a definitive statement signed by a board-certified radiologist, according to clinical routine. The primary care physician documented the preliminary CXR result in the CRF, and then decided whether or not to prescribe antibiotics. At the control units the participants were initially managed in the same way, with clinical examination and CRP testing. Decision of any referral for CXR was made by the physicians as in ‘usual care’. The physicians documented their degree of suspicion, whether or not CXR was performed, preliminary result of CXR when executed, and if any antibiotics were prescribed.

### Statistics

Clinical data and demographic characteristics of the study population were presented as proportions and median values. Medians were compared using Mann-Whitney U-test.

Pearson’s chi-square test was used for crude group comparisons and Fisher’s Exact Test for subgroup analyses when the expected number was less than 5. Data were adjusted for other variables in a multiple logistic regression model. Odds ratios were calculated with 95% confidence intervals. When data were missing, the case was left out of the analyses.

*P* values <0.05 were considered significant. Naegelkirke R square and area under curve (AUC) was estimated, with 95% confidence interval, as measures of internal validity of the multiple logistic regression models.

Statistical analyses were performed using IBM SPSS Statistics version 26 (IBM, NY, USA).

## Results

We included 104 patients in the intervention group and 81 patients in the control group. Minor differences at baseline were seen in a few patient characteristics (Table 1). In both groups, 60% of the patients were examined by GPs and the remaining by resident physicians. In the intervention group 85% were referred for CXR compared to 26% in the control group, indicating adherence to the intervention. Longer duration of symptoms was significantly associated with referral for CXR (*p* = 0.029) in the control group.

**Table 1.** Patient characteristics for anamnestic data, clinical and laboratory findings and the physician’s degree of suspicion (*n* = 185).

	Intervention		Control			
	*n* = 104		*n* = 81			
		Data		Data	Range	*p* Value
		missing		missing		
Male	53 (51%)	0	41 (51%)	0		0.96
Age (years)	56	0	49	0	18–79	0.066^a^
Current smoker	11 (11%)	3	6 (8%)	2		0.45
Antipyretics	44 (44%)	3	35 (47%)	7		0.62
Body temperature (°C)	37.3	1	37.5	2	35.4–40.0	**0.008^a^**
CRP (mg/L)	53	0	65	0	5–160	**0.026^a^**
Abnormal focal chest sound	63 (62%)	2	53 (65%)	0		0.61^a^
Symptom duration (days)	10	1	7	1		**0.002^a^**
Degree of suspicion						0.076^a^
Unsure	41 (39%)		22 (27%)			
Quite sure	35 (34%)		30 (37%)			
Sure	28 (27%)		29(36%)			
Antibiotics	71 (69%)	1	62 (77%)			0.25
Referred for CXR	88 (85%)		21 (26%)			**<0.001**

Data are presented as numbers or medians.

Pearson’s chi-square test was used for group comparison if not specified others. *P* values <0.05 were considered significant and are bolded in table.

^a^Mann–Whitney *U* test.

### Antibiotic prescribing rate

The antibiotic prescribing rate was 72% in total and did not differ between GPs and resident physicians. Among all patients (both groups) 65 years and older (*n* = 57), the prescribing rate was 83%, compared to 68% in patients <65 years (*n* = 127) (*p* = 0.039). There was no significant difference in antibiotic prescribing rate between the intervention and control group as shown in Table 2. CRP level and ‘degree of suspicion’ were the most important predictors for prescribing of antibiotics. When the same analysis was performed leaving out ‘degree of suspicion’, CRP turned out as an even stronger predictor (*p* < 0.001, OR 1.4 [CI 1.2-1.7]). No other variable was significant. Patients referred for CXR in the control group, received less antibiotics, 52% (*n* = 11), compared to those who were not, 85% (*n* = 51) (*p* = 0.005).

**Table 2.** Factors correlating to the propensity to be prescribed antibiotics when community acquired pneumonia is suspected.

	Univariable logistic regression			**Multivariable logistic regression (*n* = 166)	
	*n*	*p* Value	OR (95% CI)	*p* Value	OR (95% CI)
Intervention (1) vs. control (0)	184	0.25	0.68 (0.35–1.3)	0.81	1.1 (0.43–3.0)
Male	184	0.36	1.4 (0.71–2.6)	0.41	1.5 (0.56–3.8)
Age (per years)	184	0.59	1.0 (0.99–1.0)	0.50	0.99 (0.97–1.0)
Symptom duration (per days)	182	**0.04**	0.96 (0.93–1.0)	0.40	0.97 (0.90–1.0)
Body temperature (per ˚C)	181	**0.03**	1.7 (1.1–2.8)	0.80	1.1 (0.54–2.2)
Smoking	179	0.38	1.6 (0.56–4.6)	0.56	1.6 (0.35–7.0)
Antipyretics	174	0.71	0.88 (0.45–1.7)	0.26	1.8 (0.64–5.0)
Abnormal focal chest sound	182	0.68	0.87 (0.45–1.7)	0.93	0.96 (0.34–2.7)
Degree of suspicion*	184	**<0.001**	8.5 (4.4–16.5)	**<0.001**	6.7 (2.7–16.9)
CRP (per 10 mg/L)	184	**<0.001**	1.4 (1.2–1.5)	**0.024**	1.2 (1.03–1.4)

Crude and adjusted data (enter model). P values <0.05 were considered significant and are bolded in table.

*The physicians’s suspicion of pneumonia – unsure, quite sure, or sure.

**Naegelkerke *R* square 0.52, area under curve (AUC) 0.89 (0.84–0.94).

### Prescribing rates in relation to negative CXR

In total, the antibiotic prescribing rate for patients with negative CXR (*n* = 55) was 24%. The corresponding rate in the intervention group was 18% (*n* = 6) compared to 38% (*n* = 7) in the control group (*p* = 0.17). The most important factor for prescription of antibiotics when CXR was negative was the physician’s degree of suspicion as seen in Table 3. When running the same multiple logistic regression without ‘degree of suspicion’, no other variable was statistically significant. Men with negative CXR were prescribed antibiotics in 36% of the cases compared to 11% in women.

**Table 3.** Factors correlating to the propensity to be prescribed antibiotics when chest X-ray is negative in patients were community acquired pneumonia is suspected in primary care.

	Univariable logistic regression		**Multivariable logistic regression	
			*n* = 51	
	*p* Value	OR (95% CI)	*p* Value	OR (95% CI)
Intervention (1) vs. control (0)	0.13	0.36 (0.099–1.4)	0.18	0.27 (0.38–1.9)
Male	**0.041**	4.4 (1.1–19)	0.29	3.5 (0.35–34)
Age (per years)	**0.015**	1.1 (1.01–1.1)	0.54	1.0 (0.99–1.0)
Symptom duration (per days)	0.50	0.97 (0.90–1.1)	0.64	1.0 (0.92–1.2)
Body temperature (per ˚C)	0.18	2.1 (0.71–6.1)	0.49	0.53 (0.89–3.2)
Antipyretics	0.42	1.7 (0.46–6.6)	0.67	1.7 (0.16–18)
Abnormal focal chest sound	0.32	1.9 (0.54–6.7)	0.22	3.9 (0.44–35)
Degree of suspicion*	**0.001**	6.6 (2.2–20)	**0.02**	5.8 (1.3–26)
CRP (per 10 mg/L)	**0.001**	1.4 (1.1–1.6)	0.21	1.2 (0.9–1.6)

Crude and adjusted data (enter model). P values <0.05 were considered significant and are bolded in table

*The physicians’s suspicion of pneumonia – unsure, quite sure, or sure.

**Nagelkerke R Square 0.60, area under curve (AUC) 0.91 (0.81–1.0).

### Subgroup analyses on suspicion degree

In the intervention group, when the physician was ‘unsure’ or ‘quite sure’, 97% (*n* = 74) were referred for CXR, compared to 33% (*n* = 17) in the control group (*p* < 0.001). When the physician rated the suspicion degree as ‘unsure’, antibiotics were prescribed in 42% (*n* = 17) in the intervention group, and 32% (*n* = 7) in the control group (*p* = 0.59). The duration of symptoms differed significantly between the suspicion degrees ‘unsure’ and ‘sure’, 10 *vs* 7 days (*p* = 0.002)

## Discussion

This study could not prove that the advised routine to consistently refer all patients with suspected pneumonia for CXR, unless the physician was ‘sure’ of the clinical diagnosis of pneumonia, results in a lower antibiotic prescribing rate, compared to ordinary clinical practice.

A surprising phenomenon observed is that the physicians, to an alarming proportion, did not appear to trust the CXR outcome, but tended to prescribe antibiotics anyway, in as much as one out of four negative CXRs. However, when calculating the antibiotic prescribing rate, assuming that negative CXRs would have resulted in no antibiotic prescribing, the difference was still not statistically significant. Even if CXR is considered ‘gold standard’ we know it is an imperfect reference. It has been shown that high resolution computed tomography will find more infiltrates and may, to a higher degree, be able to differentiate between viral and bacterial infiltrates. However, this is not easy as there is a diagnostic overlap [18,19]. The physician´s preconceptions about diagnosis and treatment might play a role and reduce adherence [20]

The degree of suspicion of pneumonia was a predictor of antibiotic prescribing among patients with neg CXR, possibly indicating a higher trust in the clinical judgement than the CXR outcome.

In the control group, one out of four in total, and one out of three in cases rated as ’unsure ‘or ’quite sure‘, was referred for CXR (thus even in absence of intervention). This contributes to a less marked difference between the intervention and control group regarding diagnostic management and antibiotic prescribing rate.

The physicians’ degree of suspicion varied with symptom duration, where a higher degree of suspicion was associated with shorter duration. Also, a decision to refer patients for CXR in the control group was more common in case of longer symptom duration. This seems adequate by means of using CXR as a diagnostic tool in cases of greater uncertainty in the diagnosis of LRTI.

### Strengths and weaknesses

The intervention seemed to have been accurately implemented since 97% of the patients in the intervention group, were referred for CXR, which is a strength. Another strength is that inclusion of patients took place in two separate counties, for both the intervention and control group. This is likely to counteract local clinical routines as a potential confounder. Moreover, we only included patients with respiratory symptoms that had lasted for more than 24 h, which is also a strength, as we know that some pneumonias do not show on CXR early in the course of the disease. The prevalence of smoking, in the present study, is in line with data from the national public health agency, which makes the material reliable and representative in this aspect [21].

The circumstance that a considerable proportion of the total number of eligible patients might not have been included, probably partly due to the physician’s shortage of time, is a limitation. Another weakness is that we did not manage to reach the predetermined sample size in the control group, which contributes to lack of power. Further, there was no randomization, a procedure that would however not have been suitable since the control group then could be contaminated by means of the intervention if the physicians assessed patients in both groups. To some extent contamination yet probably occurred, as knowledge of the study itself might have acted as a reminder that CXR could help in the assessment and could be a contributing factor to the high amount of CXRs in the control group. Further, we lack information on agreement between preliminary and definitive answer from the CXRs, which is a limitation.

### Findings in relation to other studies

Contrary to the present study, Speets et al. [22] showed that referring for CXR reduced the number of antibiotic prescriptions. However, they used other inclusion criteria and did not compare to any control group. In our control group, the proportion of antibiotics prescribed was lower among those where CXR was performed as compared to those who were not referred for CXR.

Like Blaeuer et al. [23] we found that a high proportion of patients were prescribed antibiotics despite negative CXR. Other studies also report antibiotic treatment for patients diagnosed with acute bronchitis, which could be the same phenomenon if there was a diagnostic shift, but this cannot be concluded from our data [24–26]. Another explanation could be that the physicians use CXR to exclude other underlying diseases, such as cancer [27]. This may explain why it was used more often for patients with longer symptom duration. The proportion of CXRs was high in the control group as compared to a recent Swedish register-based study where CXR was shown to be used in 12% of patients with pneumonia in primary care [28].

There was no association between abnormal chest sounds and antibiotic prescribing. Other studies have shown that auscultation findings are not discriminative for pneumonia [13, 29,30], whereas some studies from primary care, have shown that crackles in combination with some other clinical findings, or alone, can be predictable for pneumonia [12, 31]. Van Vugt et al. [32] found that 71% of patients with acute cough in combination with a new infiltrate on CXR, were not clinically diagnosed as pneumonia, and that they had milder symptoms compared to patients clinically diagnosed with pneumonia. This might be the case even in our study regarding those in the intervention group with the suspicion degree ‘unsure’, who were prescribed antibiotics after CXR result. If extended use of CXR results in findings of more radiographic infiltrates, for which none of those patients would benefit from antibiotics, CXR might not be a preferable gold standard.

### Implications

The results of our study support several current European guidelines recommending not to use CXR in the initial judgement of suspected pneumonia. It is surprising that the physicians to a greater extent do not appear to trust the result of the CXR. It would be of interest to find out what the reason for this might be. A qualitative research approach, preferably based on focus group interviews, could possibly be suitable to address this question.
